# Parameterization of physical properties of layered body structure into equivalent circuit model

**DOI:** 10.1186/s42490-021-00054-8

**Published:** 2021-05-20

**Authors:** Jiho Lee, Sung-Min Park

**Affiliations:** 1 Department of Creative IT Engineering, Pohang University of Science and Technology(POSTECH), Pohang, Republic of Korea; 2 Medical Device Innovation Center, Pohang University of Science and Technology(POSTECH), Pohang, Republic of Korea; 3 Department of Electrical Engineering, Pohang University of Science and Technology(POSTECH), Pohang, Republic of Korea

**Keywords:** Equivalent circuit model, Electromagnetics, Electrical nerve stimulation, Multi-layer body impedance, Direct parameterization of conductivity, permittivity, and thickness, Layer-by-layer dynamic impedance

## Abstract

**Background:**

This study presents a novel technique to develop an equivalent circuit model (ECM) for analyzing the responses of the layered body structure to transcutaneous electrical nerve stimulation (TENS) by parameterizing electrical and geometrical properties.Many classical ECMs are non-parametric because of the difficulty in projecting intrapersonal variability in the physical properties into ECM. However, not considering the intrapersonal variability hampers patient-specifically analyzing the body response to TENS and personal optimization of TENS parameter design. To overcome this limitation, we propose a tissue property-based (TPB) approach for the direct parameterization of the physical properties in the layered body structure and thus enable to quantify the effects of intrapersonal variability.

**Results:**

The proposed method was first validated through in vitro phantom studies and then was applied in-vivo to analyze the TENS on the forearm. The TPB-ECM calculated the impedance network in the forearm and corresponding responses to TENS. In addition, the modelled impedance was in good agreement with well-known impedance properties that have been achieved empirically.

**Conclusions:**

The TPB approach uses the parameterized circuit components compared to non-parametric conventional ECMs, thus overcoming the intrapersonal variability problem of the conventional ECMs. Therefore, the TPB-ECM has a potential for widely-applicable TENS analysis and could provide impactful guidance in the TENS parameter design.

**Supplementary Information:**

The online version contains supplementary material available at (10.1186/s42490-021-00054-8).

## Background

Transcutaneous electrical nerve stimulation (TENS) has been in use as a clinical neuromodulation modality for decades. There are many FDA-approved TENS devices in the market [1–4]. While the TENS technique has shown clinically meaningful effects, the pulse parameters such as shape, intensity, and repetition rate have not been designed with optimizing for individual patients [5–8]. This is because there is lack of an effective method considering the intrapersonal variability in physical properties of the body tissue to when analyzing the body response to an externally applied electrical stimulus. Therefore, it is important to develop such a method to optimize the TENS parameters that can minimize the unwanted energy loss in the background tissues and the safety concerns such as pain and skin irritation [9–11].

The equivalent circuit model (ECM) and finite element method (FEM) have been widely accepted methods for analyzing the body response to an externally applied electrical stimulus. The FEM has significant advantages in calculating the detailed field distribution, but the ECM has been used more commonly to analyzes pulse-shaped TENS because of its low computational burden [12–15]. When the FEM analyzes the response to a general pulse-shaped stimulus, the response is typically calculated by using the summation of sinusoidal components. Thus, the response of each frequency must be calculated and summed to obtain the response due to the pulse shaped stimuli. Therefore, computing for all frequency components can be a large computational burden [16–19]. On the other hand, the ECM approach provides simple explicit solutions for each frequency, and thus is computationally efficient for computing the responses to a pulse shaped stimulus. Nonetheless, the main limitation of the conventional ECM is that it uses non-parameterized circuit components, and thus cannot be used to analyze the effects of intrapersonal variability [20, 21]. Many researchers have performed in vitro and in vivo impedance experiments to design the ECMs [15, 17, 22]. However, the proposed models differed in circuit composition and circuit component values for each researcher due to the intrapersonal variability. Due to a lack of an effective method to parameterize the effects of the variability, it has been challenging to analyze the individually accurate response to TENS and design the personally optimized TENS parameter. Thus, advances to the ECM are needed for analyzing the body responses by discovering the straightforward relationship between the body impedance and the physical tissue properties.

For parameterizing the physical tissue properties, the effects of 3D electromagnetic field distribution need to be projected into the 1D impedance elements. The main bottlenecks in this dimensional transition are the modeling of nonlinear effects of geometrical property on the impedance and the interactive effects among the electrical property of neighboring layers [23–26]. To overcome these challenges and develop the ECM with the physical parameters, we propose a novel tissue-property-based (TPB) method, which utilizes the hybrid techniques of computation and analytics. We modelled each tissue layer impedance as a complex capacitor parameterizing each layer’s effective permittivity, which in turn is affected by the corresponding underlying layer. This interaction between the neighboring layers can’t be modelled by existing ECM methods. Then, the network connection among capacitors was derived with the partial capacitance (PC) method [25]. We finally complete the model by computationally parameterizing the effect of geometrical tissue properties. We validated the developed model through in vitro experiments and then applied the model in-vivo to analyze the TENS on the forearm.

## Results

### ECM developed with TPB approach

**Fig. 1:**
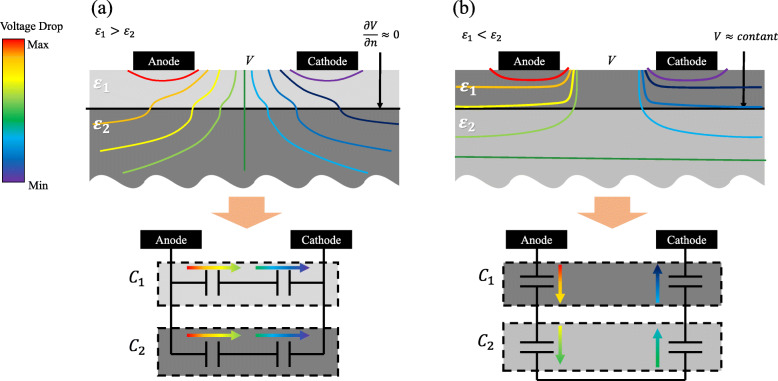
Regression for interdimensional factor (IF) based on in-silico experiments. **a**&**d** PPC and SPC IF of Layer1 versus permittivity ratio between Layer2 and Layer1 for three Layer1 thickness 2, 4, and 6 mm. Black lines are regressed model and colored dots are experimental data. **b**&**e** PPC and SPC IF of Layer1 versus Layer1 thickness versus Layer1 thickness for three permittivity settings. Solid, dashed, and dotted black curves are regressed model for each permittivity setting and colored dots are experimental data. **c**&**f** PPC and SPC IF of Layer1 for ten permittivity pairs of Layer1 and Layer2 and three Layer1 thicknesses. Black squares are regressed model and colored dots are experimental data

The 2D-to-3D expansion of PC method showed its reliability by verifying the GF terms are dominantly affected by tissue layer thickness, the geometrical tissue property (Fig. S2). The coefficients of the regression models of PPC and SPC IFs were calculated by fitting to the 3D simulation data (Fig. 1). The determined coefficients for Eqs. (5) and (6) were: (*a*_0_,*a*_1_,*a*_2_)=10^−^3(4.69,1.12,4.40) for PPC; (*b*_0_,*b*_1_,*c*_0_)=10^3^(2.83,1.00,1.30) for SPC. Consequently, the TPB model was developed by the analytical PC principles complemented by the computational regression of IFs.

### TPB-ECM agreeing to in vitro experiments

**Fig. 2:**
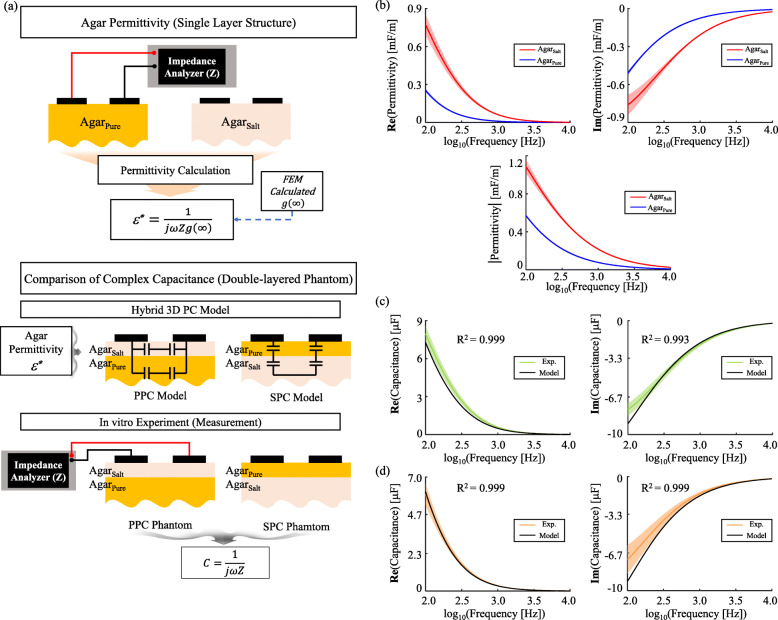
Complex permittivity measurement of salted and pure agar material and comparison of phantom capacitances. **a** magnitude of the complex permittivities, **b** phase of the complex permittivities, **c** PPC phantom capacitance comparison, and **d** SPC phantom capacitance comparison. (mean ±2 std for b, c, and d)

The modelled capacitances in ECM constructed using the TPB approach well-agreed to the in vitro results. The capacitances of double-layered agar phantoms were calculated by the developed model using the measured permittivity of Agar _*Salt*_ and Agar _*Pure*_ (Fig. 2b). The magnitude of permittivities verified that the PPC phantom was Agar _*Salt*_-on-Agar _*Pure*_, and the SPC phantom was Agar _*Pure*_-on-Agar _*Salt*_ due to the permittivity increase with the injected NaCl ion in Agar _*Salt*_. For both PPC and SPC phantoms, the real part of measured capacitances agreed well with the modelled values, but errors were present in the low-frequency components of imaginary parts (Fig. 2c and d). Except for the low-frequency imaginary error, there were the overall tendency matches between the model and the experiment with each correlation coefficient over 0.9.

### Forearm impedance and response to TENS analyzed with TPB-ECM

**Fig. 3:**
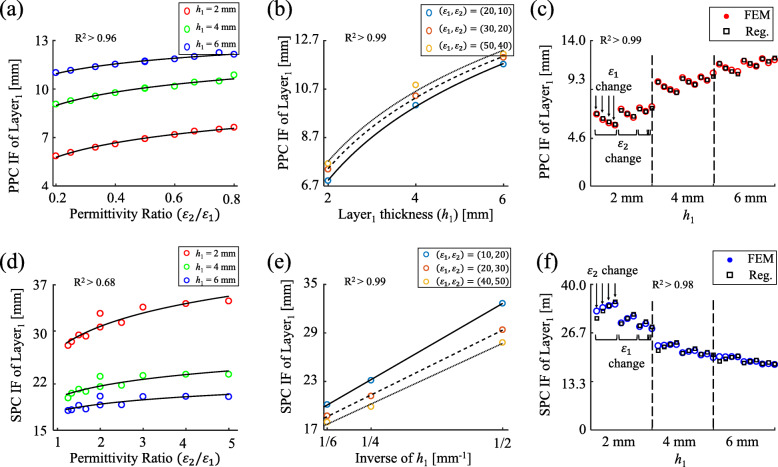
The forearm complex permittivity analysis, the impedance, and the effects of tissue property variance on the impedance. **a** the complex permittivity for skin, fat, and muscle, **b** layer-by-layer impedance (left) and the total impedance (right) with the skin thickness 1.5 mm and the fat of 2.5 mm (muscle, musch thicker than the skin and fat was considered as virtual infinite layer), **c** the total impedance changes due to skin (top raw) and fat (bottom raw) thicknesses from 1 to 3 mm, **d** the total impedance changes due to skin, fat and muscle permittivities from -20 to 20% variable

We applied the TPB-ECM to analyze the multilayered forearm structure. The impedance network of the forearm and its electrical responses to square-pulse TENS were calculated. Permittivities of forearm tissue structure satisfied the SPC condition (|*ε*_*muscle*_|>|*ε*_*fat*_|>|*ε*_*skin*_|) (Fig. 3a). Compared to the skin impedance (≈30 k *Ω*), the impedance magnitude of fat (≈1 k *Ω*) and muscle (≈ 0.1 k *Ω*) layers were relatively small. Thus, the total impedance followed the skin impedance tendency as expected from the references of empirical measurements [27] (Fig. 3b). In addition, the analysis of permittivities with varying tissue layer thicknesses clearly showed that the skin thickness was the dominant factor for total impedance compared to other variables. The skin thickness changed the total impedance from 30 to 80 k *Ω*, whereas the changes of the fat thickness and each permittivity of skin, fat, and muscle did not affect the total impedance significantly (Fig. 3c and d).

**Fig. 4:**
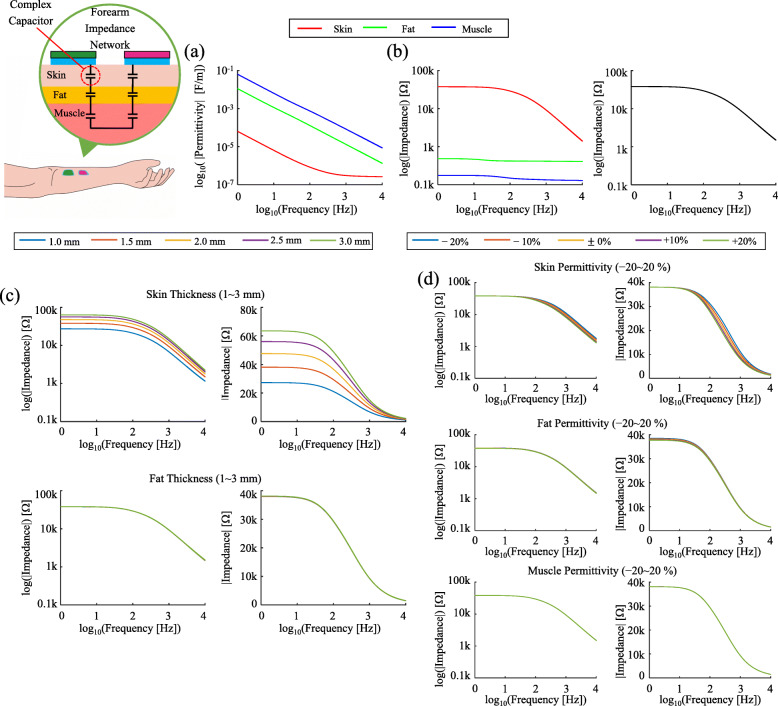
Electrical responses of the forearm impedance network to voltage-driven pulse stimulation and expected current change according to pulse width (W) and stimulation frequency (F) of the voltage-driven stimulation input. **a** voltage-driven pulse signal (intensity 1 [V]/ pulse width 3 [ms]/ stimulation frequency 10 [Hz]), **b** current response to the stimulation, **c** loaded voltage on skin layer, **d** loaded voltage on fat layer, **e** loaded voltage on muscle layer, **f** the current change versus the stimulation frequency change (W=3 [ms]), **g** the current change versus the pulse width change (F=10 [Hz]), **h** total map of the current change

When a square-wave voltage pulse was applied to the impedance network, a distorted current pulse was induced through the serially connected complex capacitor network (Fig. 4a and b). The shape and intensity of voltage loaded on the skin were similar to the source, whereas the voltages loaded on the fat and muscle were very small and shaped similarly to the total current (Fig. 4c-e). The variance in pulse width significantly changed the amount of total current (from 100 to 30 *μ*A), but the stimulation frequency did not induce significant change (only from 30 to 20 *μ*A) (Fig. 4f-h). Thus, even though the large drop of the supplied voltage near the skin resulted in tiny current flowing into the body, it was possible to increase the current penetrating into the body dominantly by adjusting the pulse width. Similarly, analyses of the forearm responses to current-driven TENS showed that a high voltage (≈ 40 V) was required to flow the unit current inside the body (1 mA). However, the current source could also avoid the effect of high skin impedance and thus reduce the high voltage load at the source by decreasing the stimulation frequency and increasing the pulse width (Fig. S1).

## Discussion

The ECM is very useful in modeling the impedance of a body segment and in analyzing the response to TENS. It calculates the comprehensive response to multi-frequency components of pulsed-TENS better than other methods such as the numerical volume conductor approach. However, the current ECMs use non-parameterized circuit components thereby not including the effects of intrapersonal variability in physical tissue properties such as thickness and electrical properties. This hinders personal optimization of TENS parameter design for each patient in terms of minimizing penetration energy and skin damage.

In this study, we designed a new approach for modeling the body impedance network, named as the TPB, to directly parameterize the physical properties into the ECM. The TPB approach employs a hybrid technique and incorporates both analytic and computational methods to overcome the difficulty of projecting 3D electromagnetic field distribution into a 1D equivalent circuit. The TPB models effects of both electrical and geometrical properties. The analytic PC method can even address the interaction of complex permittivity between neighboring layers and the connection between their complex capacitors. The 3D FEM simulation complements the PC method equations by parameterizing the nonlinear layer-by-layer geometrical properties.

The developed TPB-ECM was validated through in vitro experiments on phantoms. The real and imaginary components of the measured complex capacitances in phantoms were compared against the modelled values. In other words, the model was verified by evaluating both the capacitive and resistive components of the phantom’s impedance. The results showed that the model generally agreed well with the measurement. At low frequency, there were underestimations in imaginary parts, which implies overestimation in the modelled conductivity. This error is most likely due to electrode-skin impedance being high at low frequency [28, 29], which was not considered in this study.

Finally, to evaluate the model’s usefulness in-vivo, we applied the TPB-ECM to multilayered forearm structure and compared them to well-known reference data, which have been derived empirically. The modelled and measured on-skin impedance similarly showed the RC low pass filtering with 100 Hz cur-off and about 30 to 80 k *Ω* of maximum at low frequency. Among the physical parameters, the skin thickness had more influence on impedance than other parameters. These findings agree well with the known experimental references [30, 31]. In addition, the variance of modelled impedance by the change of skin thickness were similar with the perosonal variabnce of measured impedance shown in the reference data, this implies that the intrapersonal variability can be quantitatively modelled by the proposed TPB-ECM unlike the conventional ECMs. Similar to the in vitro error, the modelled low-frequency impedances were relatively lower than the reference, mainly due to the electrode-skin interfacing impedance. By adding the conventional electrolyte-skin impedance model, the modelled impedance of TPB-ECM can be made to have high accuracy in both low frequency as well as high frequency. Using the network of variable impedances in forearm, the forearm responses were analyzed using the conventional square pulse TENS. The applied source voltage was largely consumed at the skin surface. By shortening pulse width, the voltage drop at the skin surface can be mitigated, which is consistent with the reported experimental results [8, 20, 32]. These results validate the TPB-ECM method, showing that the TPB-ECM can include the effects of intrapersonal variability in physical tissue properties, and thus has a potential to be applied to personally optimize the TENS parameter design.

## Conclusion

The TPB-ECM approach proposed here to analyze the response to TENS provides a method for direct physical property parameterization, including the electrical interaction between adjacent layers of body structures and the nonlinear geometrical effects of each layer. The TPB-ECM was based on the PC method with the concept of complex capacitor. The complex capacitor can integrate the resistance and capacitance into a single component, and thus have a one-to-one correspondence with the impedance of each tissue layer. Compared to the conventional ECMs, TPB-ECM avoids the risk of inaccuracy caused by the intrapersonal variability in physical tissue properties and thus can personally optimize the analysis of the response to TENS and the TENS parameter design. The TPB-ECM results agree well with the experimental results in in vitro experiments and with in-vivo results derived empirically. For the practical application of the developed TPB-ECM, one maybe obtain the physcial tissue properties with the minimally invasive and pain free needles for electrical properties and the noninvasive medical imaging such as ultrasound for geometrical properties [33]. We believe, TPB-ECM has the potential to become a commonly used platform to analyze the response to TENS and could provide impactful guidance in the standardization of TENS parameter design.

## Methods

### Analytical method to parameterize the effective electrical property into ECM

Due to the interactions among the neighboring layers, the effective electrical properties of each layer are different from their own properties. The following section describes analytical processes for parameterizing the effective electrical properties and importing them into ECM.

#### Complex permittivity to simplify the electrical property of a tissue layer

The complex permittivity simplifies two representative electrical properties of conductivity and permittivity analytically ($\varepsilon ^{*} = \varepsilon -j\frac {\sigma }{\omega }$, where *ε*^∗^ is complex permittivity, *ε* is real permittivity, *σ* is conductivity, and *ω* is angular frequency) into a single property. Correspondingly, the RC impedances of a tissue layer can be simplified into a single complex capacitor. The impedance is given by:

1 $$ \begin{aligned} Z_{tot} & = R || \frac{1}{j\omega C}\\ \therefore{} & = \frac{1}{j \omega \left(\varepsilon - j \frac{\sigma}{\omega}\right) \frac{A}{d}} = \frac{1}{j \omega \varepsilon^{*} \frac{A}{d}} = \frac{1}{j \omega C^{*}}\,. \end{aligned}  $$

where *R*(=*d*/*σA*, *A* is the area, and d is the distance between source electrodes) is the resistance, *C*(=*εA*/*d*) is the real capacitance, and *C*^∗^(=*ε*^∗^*A*/*d*) is the complex capacitance. Therefore, the complex capacitor with the complex permittivity can simultaneously include both of the resistance and the capacitance parts. This simplification enables the application of an analytical method, called the partial capacitance. The method can model the effective complex permittivity of each tissue layer where the interactions among neighboring layers are considered.

#### Partial capacitance method to parameterize the effective complex permittivity into capacitor network

**Fig. 5:**
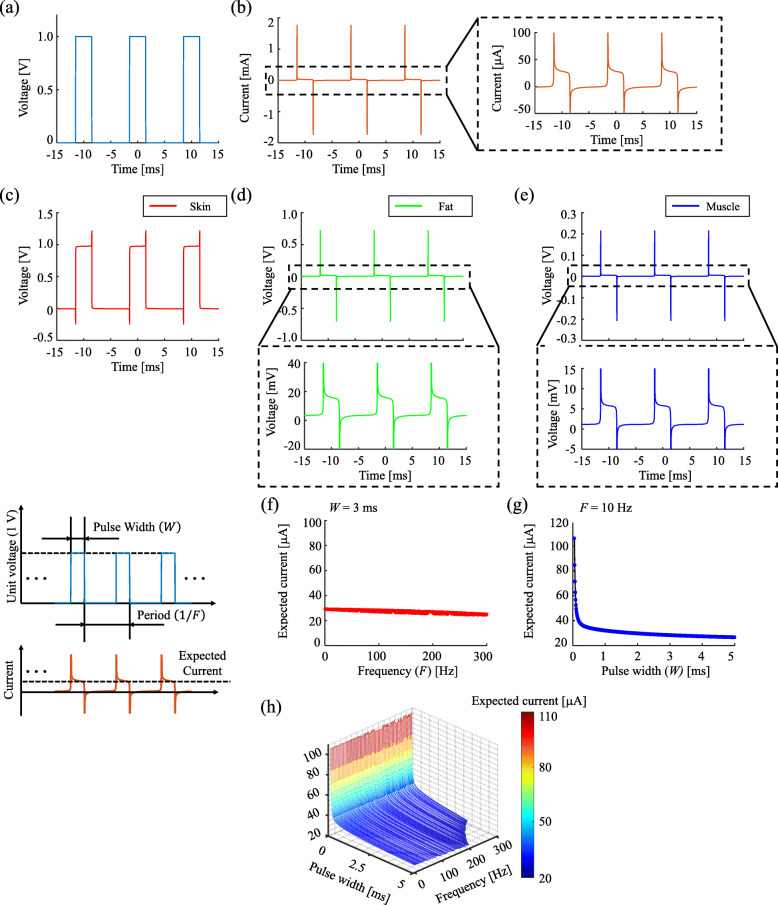
Principle of PC method. **a** Parallel partial capacitance (PPC) for *ε*_1_>*ε*_2_ has Neumann boundary condition between layers and thus is modelled as capacitors in parallel connection, **b** Serial partial capacitance (SPC) *ε*_1_<*ε*_2_ Dirichlet boundary condition between layers and thus is modelled as capacitors in serial connection

The partial capacitance (PC) method decomposes the total effective capacitance of the layered structure as the sum of each layer capacitance [23–25]. This physically models a capacitor network of the structure with layer-by-layer partial capacitors. For each partial capacitor, the PC method decides its direction, whether parallel-to-layer (current shunting along with the layer) or serial-to-layer (current penetrating through the layer) (Fig. 5). The direction of a layer capacitor is dependent on the permittivity ratio between the layer and its underlying layer. In the first category, the parallel partial capacitance (PPC) condition is the case when the upper layer (Layer_1_) has a higher permittivity than the lower layer (Layer_2_) with layer interface under Neumann boundary condition (Fig. 5a) [23]. The PPC for the total capacitance is defined as:

2 $$ \begin{aligned} C_{tot} & = C_{1} + C_{2}\\ & = \left(\varepsilon_{1} - \varepsilon_{2}\right)g_{1} + \varepsilon_{2} g_{2},\quad \left(\varepsilon_{1}>\varepsilon_{2}\right) \end{aligned}  $$

where C _*tot*_ is the total capacitance, *C*_*i*_ is the Layer _*i*_ capacitance *ε*_*i*_ is the Layer _*i*_ permittivity, and *g*_*i*_ is PPC geometric factor of Layer _*i*_. The geometric factor (GF) of each layer is defined as:

3 $$ \begin{aligned} g\left(x_{1}, x_{2}, x_{3}, \ldots\right)=\frac{C}{\varepsilon} \end{aligned}  $$

where *x*_*i*_ is the geometric variables, such as the thickness of layer, the electrode size, and the distance between electrodes. In the second category, serial partial capacitance (SPC) is the case when Layer_1_ has a lower permittivity than Layer_2_ with satisfying the interface under Dirichlet boundary condition (Fig. 5b). The SPC for the total capacitance is defined as:

4 $$ \begin{aligned} \frac{1}{C_{tot}} &= \frac{1}{C_{1}}+\frac{1}{C_{2}}\\ &= \left(\frac{1}{\varepsilon_{1}} - \frac{1}{\varepsilon_{2}} \right) \frac{1}{{g_{1}}^{'}} + \frac{1}{\varepsilon_{2} {g_{2}}^{'}}, \quad \left(\varepsilon_{1}<\varepsilon_{2}\right) \end{aligned}  $$

where $\phantom {\dot {i}\!}{g_{i}}^{'}$ is SPC GF of Layer _*i*_, and the other variables are similar to PPC formula. Moreover, prior research has demonstrated that the approach is valid when using the complex capacitors [25]. The PC method formulas in Eqs. (2) and (4) imply that the effective (complex) permittivity of Layer_1_, which is affected by Layer_2_, can be described as *ε*_1_−*ε*_2_ for PPC and 1**/***ε*_1_−1**/***ε*_2_ for SPC. The method also describes the connection information between the capacitors of two neighboring layers, and thus parameterizes the effective complex permittivity of each layer to the ECM network consisting of complex capacitors.

### Computational method to parameterize the effects of geometrical property

To parameterize the remaining effects of geometrical properties into the PC method formulas in Eqs. (2) and (4), 3D electromagnetic simulations were utilized because the pure analytical derivation of the 3D GF terms requires redundantly complicate processes (the electric field distribution follows the elliptic functions and their integral would be almost impossible). Therefore, to avoid unnecessary complexity but complete the TPB-ECM, we used regression techniques on 3D simulation results to find the nonlinear geometrical effects. The detailed idea and process are the following.

#### Interdimensional factor (IF): modified GF for error complement during the 3D expansion of PC method

Our preliminary results showed that the GF terms have secondary fluctuation errors when expanding the original 2D PC equations to 3D (see Supplementary Section 1). Even though the errors are secondary effects, they still reduce the accuracy of the 3D PC equations. To eliminate the errors and increase the accuracy of the TPB model equations, a concept of modified GF was newly defined and named as an interdimensional factor (IF). The IFs are assumed to be affected by both Layer_1_ thickness (*h*_1_) and the permittivities ratio between Layer_1_ and Layer_2_ (*ε*_2_/*ε*_1_) because the fluctuation errors in 2D-to-3D expansion of PC method depend on the layer permittivities (see Supplementary Section 2).

#### Regression for IF and final TPB model equations

3D simulation data with the inputs of (*h*_1_,*ε*_1_,*ε*_2_) and the output of (*C*_*PPC*_ or *C*_*SPC*_) were applied to the regression model to formulate IFs. Using the formulas of 2D Layer_1_ GF $\left (\text {PPC:}\frac {C_{PPC}-\varepsilon _{2} g_{2}}{\varepsilon _{1} - \varepsilon _{2}}, \text {SPC:}\frac {1\mathbf {/}\varepsilon _{2} -1\mathbf {/}\varepsilon _{1}}{1\mathbf {/}C_{SPC} -1\mathbf {/}\varepsilon _{2} g_{2}}\right)$, the regressions were performed for each of PPC IF and SPC IF. For PPC IF, the logarithmic regressions were performed and the final PPC model is described as:

5 $$ \begin{aligned} C_{PPC}^{3D} &= \left(\varepsilon_{1} - \varepsilon_{2}\right) I_{PPC}^{3D} + \varepsilon_{2} g_{2} \\ with \quad I_{PPC}^{3D} &= a_{0} + a_{1} \ln{\left(\varepsilon_{2} - \varepsilon_{1} \right)} + a_{2} \ln{(h_{1})} \end{aligned}  $$

where $I_{PPC}^{3D}$ is the PPC IF and *a*_*i*_ (*i*=0, 1, 2) are regression coefficients. For SPC IF, inverse-linear regression on *h*_1_ and the logarithmic regression on *ε*_2_/ *ε*_1_ were performed. Then the final SPC model is described as:

6 $$ \begin{aligned} \frac{1}{C_{SPC}^{3D}} &= \left(\frac{1}{\varepsilon_{1}} - \frac{1}{\varepsilon_{2}} \right) \frac{1}{I_{SPC}^{3D}} + \frac{1}{\varepsilon_{2} g_{2}} \\ with \quad I_{SPC}^{3D} &= \frac{b_{0} + b_{1} \ln{\bigl(\frac{\varepsilon_{2}}{\varepsilon_{1}} \bigr)}}{h_{1}} + c_{0} \end{aligned}  $$

where $I_{SPC}^{3D}$ is the SPC IF and *b*_*i*_ (*i*=0, 1) and *c*_0_ are regression coefficients. All regressions used the least-square method to calculate the coefficients.

As for the simulation environment, a FEM simulator (Electrostatic solver, Maxwell 3D, ANSYS, Ansys Inc.) calculated the outputs for double-layered 3D virtual phantoms. The *h*_1_ varied from 2 to 6 mm in 2 mm-step. The Layer_2_ thickness was 50 mm, which is about 10 times thicker than Layer_1_ and enables to consider the Layer_2_ to be an infinitely thick layer. We confirmed the validity of this assumption through simulation [34, 35]. The (*ε*_1_,*ε*_2_) were in 10 different pairs for each of PPC and SPC (Table 1). In addition, the simulation had an area of 1 cm x 1 cm and were separated by a distance.

**Table 1:** Relative permittivity (*ε*_1_,*ε*_2_) pairs for 3D simulation

For PPC		*ε*_2_			
(*ε*_1_>*ε*_2_)		10	20	30	40
*ε*_1_	20	(20, 10)			
	30	(30, 10)	(30, 20)		
	40	(40, 10)	(40, 20)	(40, 30)	
	50	(50, 10)	(50, 20)	(50, 30)	(50, 40)
**For PPC**		*ε*_2_			
(*ε*_1_<*ε*_2_)		20	30	40	50
*ε*_1_	10	(10, 20)	(10, 30)	(10, 40)	(10, 50)
	20		(20, 30)	(20, 40)	(20, 50)
	30			(30, 40)	(30, 50)
	40				(40, 50)

### In vitro validation of the TPB-ECM

The PC method has a recurrent property which sequentially integrates two adjacent layers into a single layer from top to bottom layer [36]. For example, the method is applied between the first and second layer and derives an integrated layer, and then is re-applied between the integrated layer and the third layer. So, we could validate the concept simply with double-layered phantom. In vitro experiments using double-layered agar phantoms were performed to validate the TPB model. The modelled capacitances of the agar phantoms were compared to the measured values (Fig. 2a).

#### Agar fabrication and complex permittivity measurement

To prepare phantom, agar powder (BD Difco Plate Count Agar, Fisher Scientific) was first mixed with DI water at a concentration of 23.5 g/L. Two different types of agar were used to construct the double-layrered phantoms. One type (Agar _*Salt*_) used DI water with 10 g/L NaCl (Sodium Chloride, Sigma-Aldrich), and the second type (Agar _*Pure*_) used pure DI water, For Agar _*Salt*_, the conductive ions of NaCl increased the conductivity [37], thus resulting in higher complex permittivity compared to Agar _*Pure*_. The mixture was then heated on a hotplate (MSH-20D, DAIHAN Scientific) to 80-90 celsius degrees to dissolve the powder. The heated solution was poured into a mold (glass, diamter 10 cm) and was cooled for more than 3 h to make it hard.

Complex permittivities of Agar _*Pure*_ and Agar _*Salt*_ were measured to use as inputs to the TPB model. Impedances of single layer structures of each agar (cylinders, 5 cm radius, 5cm thickness) were measured using an impedance analyzer (E4990A, Keysight Technology). The measurement ranged from 0.1 to 10 kHz in a log scale with 201 samples in between. Using Eq. (3), The complex permittivities were calculated for IF of structures with the FEM simulator. The measurement electrodes were 1 cm width x 1cm length, separated by 1 cm, and slightly coated with conductive gel (Spectra 360, Parker lab.).

#### In vitro experiments: complex capacitance of double-layered agar phantom

The developed TPB model was validated by comparing the total capacitances of the modelled and the measured double-layered agar phantoms for both PPC and SPC. The phantoms were cylindrical, and their dimensions were 5cm in radius, and 2 mm and 5 cm in thicknesses for Layer_1_ and Layer_2_, respectively (Fig. S3). The experimental capacitances were calculated with the measured impedance using Eq. (1). The modelled capacitances of the phantoms were calculated using the measured agar permittivities with Eqs. (5) and (6). Both the real and imaginary parts of the complex capacitances were compared to verify that both resistive and capacitive properties of the modelled capacitances agreed well with the experiments.

### Representative application of TPB-ECM on forearm

As a representative application, in the forearm the impedance network was analyzed when the tissue properties were variable and the responses to TENS were also analyzed when the TENS pulse parameters were variable.

#### Impedance network analysis in forearm

The forearm tissue layers were classified into three categories: 1) skin, 2) fat, 3) muscle. The variances in the electrical and geometrical properties were applied: thicknesses of 1 to 3 mm in 0.5 mm-step for skin and fat (muscle was assumed to be infinite); permittivities of −20*%* to +20*%* in 10%-step for each tissue. The referred complex permittivities (±0*%*) were set as the standard dataset [38]. Moreover, the conductive gel impedance was assumed as 160 *Ω* and was added to the network (preliminary measured value not presented).

#### Response to TENS in the forearm

The electrical responses of each layer in the forearm were analyzed based on their response to square pulse TENS using the calculated network. A voltage pulse (or current) to drive stimulation was used: square wave of intensity 1 V (or 1 mA) / pulse width 3 ms / stimulation frequency 10 Hz / ramping time 1 *μ*s. Since the pulse signals have multiple frequency components, the electrical responses, such as the current flowing through the network, were calculated in the frequency domain. The response analysis was completed in the time domain after the inverse FFT. In addition, the change in total current was investigated by the changing pulse width (0.05 to 5 ms) and stimulation frequency (1 to 300 Hz).

## Supplementary Information

**Additional file 1** Supplementary script. The supplementary script describes the in-silico experiments verifying the expansion of the original 2D PC principles into 3D condition. The results show the indicator dependent on geometrical property in 2D is also dependent on the geometrical property in 3D (Fig. S2).

**Additional file 2** Figure S1. Electrical responses of the forearm impedance network to current-driven pulse stimulation. Using the developed TPB-ECM, the forearm impedance network and electrical responses to current-driven TENS were analyzed.

**Additional file 3** Figure S2. GF indicator calculated from in silico experiments using FEM simulator. a) PPC GF indicator, b) SPC GF indicator.

**Additional file 4** Figure S3. Pictures of in vitro agar experiments for TPB-ECM validation. a) &b) The double-layered agar phantom, c) representative view of the placement of measurement electrodes on the double-layered agar phantom.

## Data Availability

The datasets used and/or analysed during the current study are available from the corresponding author on reasonable request. Declarations

## Abbreviations

TENS: Transcutaneous electrical nerve stimulation
ECM: Equivalent circuit model
TPB: Tissue-property base
PC: Partial capacitance
GF: Geometric factor
PPC: Parallel partial capacitance
SPC: Serial partial capacitance
IF: Interdimentional factor
