# Supplementary material for: Parameterization of physical properties of layered body structure into equivalent circuit model
Source: BMC Biomed Eng. 2021 May 20;3:9. doi: 10.1186/s42490-021-00054-8 (PMC8139009; doi:10.1186/s42490-021-00054-8)
Supplement: Supplementary file 1 — Additional file 1 Supplementary script. The supplementary script describes the in-silico experiments verifying the expansion of the original 2D PC principles into 3D condition. The results show the indicator dependent on geometrical property in 2D is also dependent on the geometrical property in 3D (Fig. S2). [file 42490_2021_54_MOESM1_ESM.pdf]

Supplementary

Evaluation for PC method approximately expanded to 3D condition

The PC method is based on the conformal mapping theory using Shwarz-Chirstoffel transformation [1]. According to Liouville's theorem, the original PC method principles are restricted to 2D condition [2]. In here, the widely-used square shape electrodes for the TENS include the 3D fringing field at their edges, so the PC method cannot be applied originally. However, the fringing field at the narrow edges can be considered as a kind of secondary effect for the capacitance between the electrodes. This expects the original 2D PC principles would be affordably maintained even in 3D although the detailed equations should be adjusted for the geometrical tissue property terms. Thus, we hypothesized that the PC method equations could be plausibly applied to construct the capacitor network in 3D layered body structure.

1. Indicator to evaluate 3D expansion hypothesis

To evaluate the hypothesis, customized indicators were defined as inversely calculated GF from capacitance and permittivity based on the PC principles. We verified whether the GF indicators depend only on the change of geometric parameter as the principles designate. The 3D PPC indicator formulated with the original 2D PC equations (equation (2) and (4) in the manuscript) is given by:

$$\begin{aligned} C_1 &= (\varepsilon_1 - \varepsilon_1)g(h_a) + \varepsilon_1 g(\infty) \\ C_2 &= (\varepsilon_1 - \varepsilon_1)g(h_b) + \varepsilon_1 g(\infty) \\ \therefore g_{indc}(h_1, h_2) &= g(h_a) - g(h_b) = \frac{C_1 - C_2}{\varepsilon_1 - \varepsilon_2} \end{aligned} \tag{1}$$

and 3D SPC indicator is given by:

$$\begin{aligned} \frac{1}{C_1} &= \left(\frac{1}{\varepsilon_1} - \frac{1}{\varepsilon_2}\right)\frac{1}{g_1^{'}(h_a)} + \frac{1}{\varepsilon_2 g(\infty)} \\ \frac{1}{C_2} &= \left(\frac{1}{\varepsilon_1} - \frac{1}{\varepsilon_2}\right)\frac{1}{g_1^{'}(h_b)} + \frac{1}{\varepsilon_2 g(\infty)} \\ \therefore g_{indc}^{'}(h_a, h_b) &= \frac{1}{g_1^{'}(h_a)} - \frac{1}{g_1^{'}(h_b)} = \frac{\varepsilon_1\varepsilon_2\big(C_2 - C_1\big)}{C_1 C_2\big(\varepsilon_2 - \varepsilon_1\big)} \end{aligned} \tag{2}$$

where $g(h)$ is the PPC GF of Layer1 with thickness h, $g^{'}(h)$ is the SPC GF of Layer_1 with thickness h, $g(\infty)$ is the GF of Layer_2 with infinite thickness, and ε_i is the permittivity of $Layer_i$. The GF indicators are the subtractions between two different GFs of Layer_1. Equation (1) and (2) do suggest that the PC principles provide inverse calculations for the GF indicators from the FEM-calculated capacitance and permittivity. If the hypothesis is valid, the calculated GF indicators would depend dominantly on the Layer_1 thickness pair $\left(h_a, h_b\right)$. Thus, we evaluated the hypothesis by investigating whether the GF indicators under the assumptions of PC principles satisfy the GF dependency on the geometric layer thickness. As we hypothesized, the GF indicators in both of PPC and SPC conditions were dominantly affected by the thicknesses rather than permittivities. In other words, the 2D PC equations can be expanded to 3D condition.

2. In silico experiments: GF indicator in double-layered virtual phantom

The in silico experiments calculated the GF indicators between two double-layered virtual phantom A and B (Fig. S2) using a FEM simulator (Electrostatic solver, Maxwell 3D, ANSYS, Ansys Inc.). The two phantoms had different Layer1 thicknesses (h_a, h_b) but same Layer1 and Layer2 permittivity pair $(\varepsilon_1, \varepsilon_2)$. The (h_a, h_b) were varied: (2, 4), (4, 6), and (2, 6) mm. For each thickness pair, 20 different $(\varepsilon_1, \varepsilon_2)$ were simulated (Table 1 in the manuscript): one half were for PPC GF indicators using equation (1) and the other half for SPC GF indicators using equation (2). In addition, the size of the simulated electrodes was 1 cm x 1cm, the distance between electrodes was 1 cm, and the Layer2 thickness was 5 cm enough to assume infinity.

References

[1] Wang, W., Ma, W., Wang, Q., Ren, H.: Conformal mapping for multiple terminals. Scientific reports **6**, 36918 (2016)

[2] Ahlfors, L., Bers, L.: Riemann's mapping theorem for variable metrics. Annals of Mathematics, 385–404 (1960)

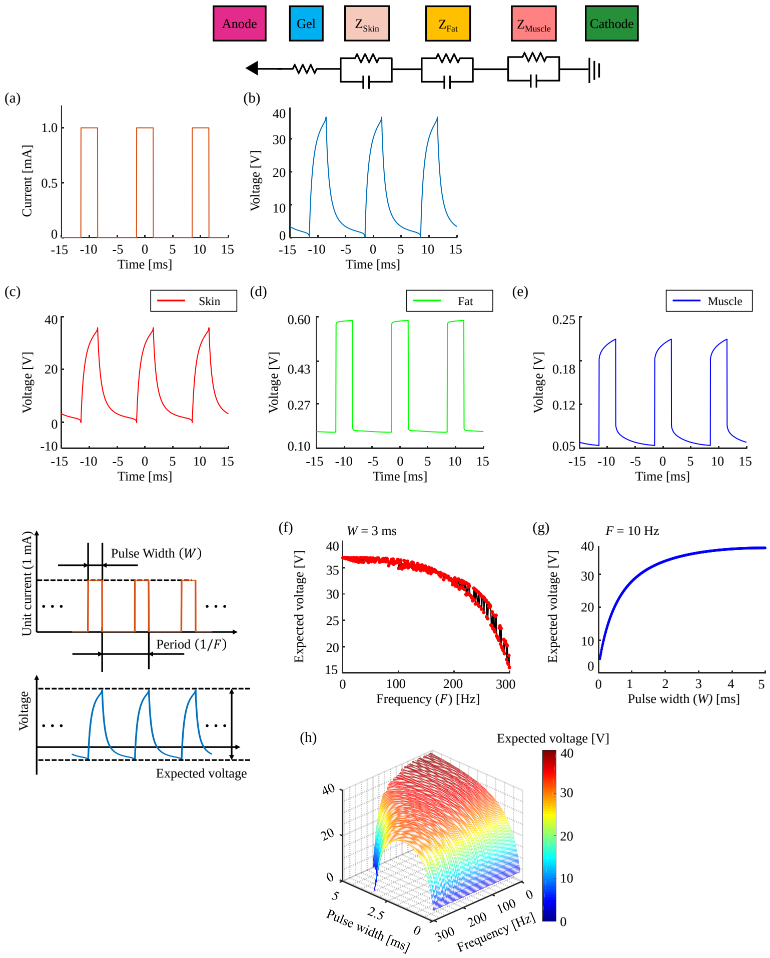

Figure S1. Electrical responses of the forearm impedance network to current-driven pulse stimulation and expected voltage change according to pulse width (W) and stimulation frequency (F) of the current-driven stimulation input. a) current-driven pulse signal (intensity 1 [ms]/ pulse width 3 [ms]/ stimulation frequency 10 [Hz]), b) overall voltage response to the stimulation, c) loaded voltage on skin layer, d) loaded voltage on fat layer, e) loaded voltage on muscle layer, f) the expected voltage versus the stimulation frequency change (W=3 [ms]), g) the voltage versus the pulse width change (F = 10 [Hz]), and h) total map of the voltage change.

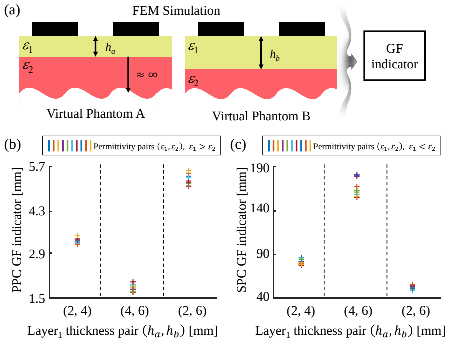

Figure S2. GF indicator calculated from in silico experiments using FEM simulator. a) PPC GF indicator, b) SPC GF indicator.

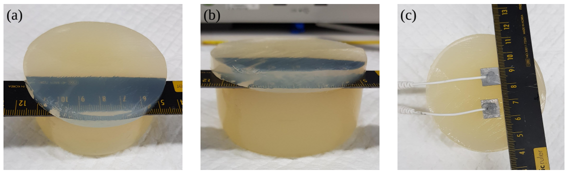

Figure S3. Pictures of in vitro agar experiments for TPB-ECM validation. a)&b) The double-layered agar phantom, c) representative view of the placement of measurement electrodes on the double-layered agar phantom.
