# Supplementary figures and images for: Parameterization of physical properties of layered body structure into equivalent circuit model
Source: BMC Biomed Eng. 2021 May 20;3:9. doi: 10.1186/s42490-021-00054-8 (PMC8139009; doi:10.1186/s42490-021-00054-8)

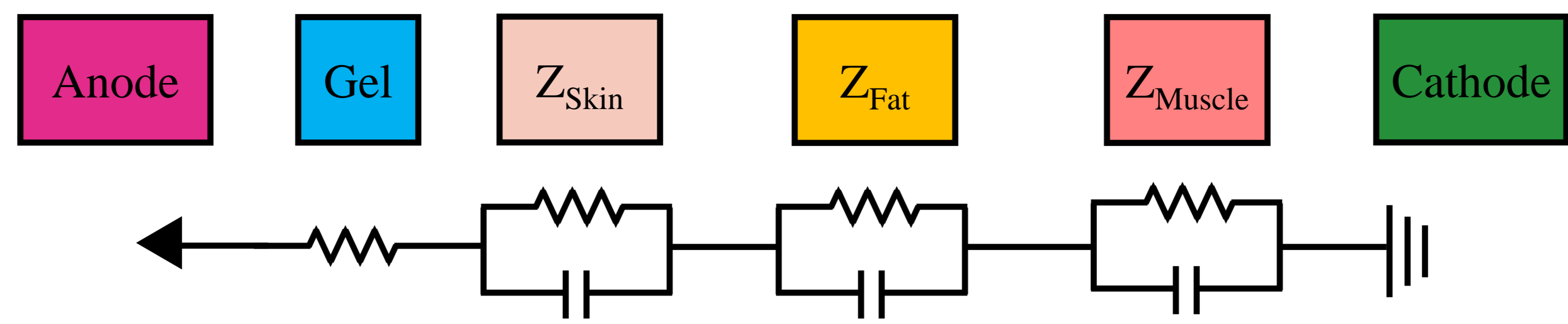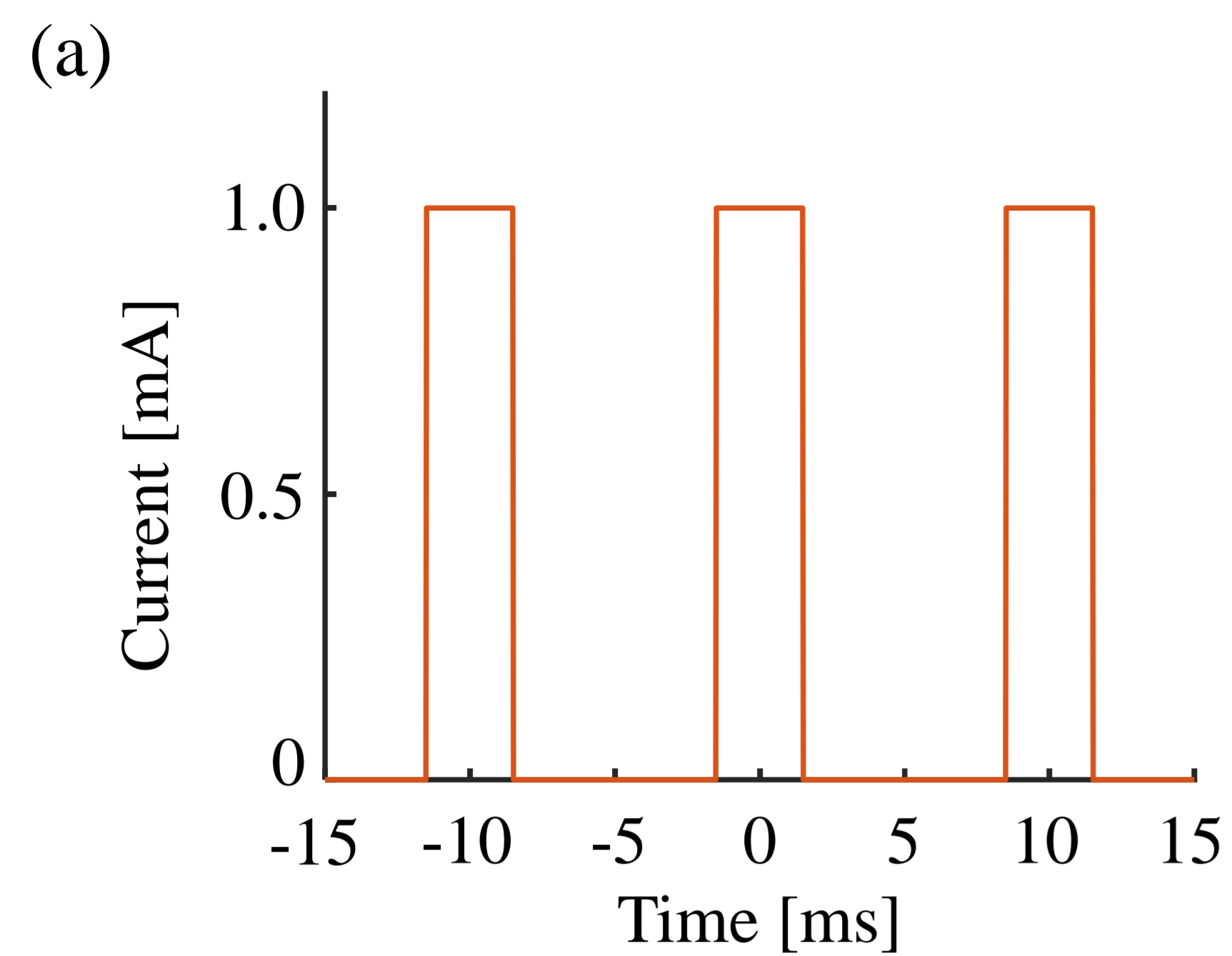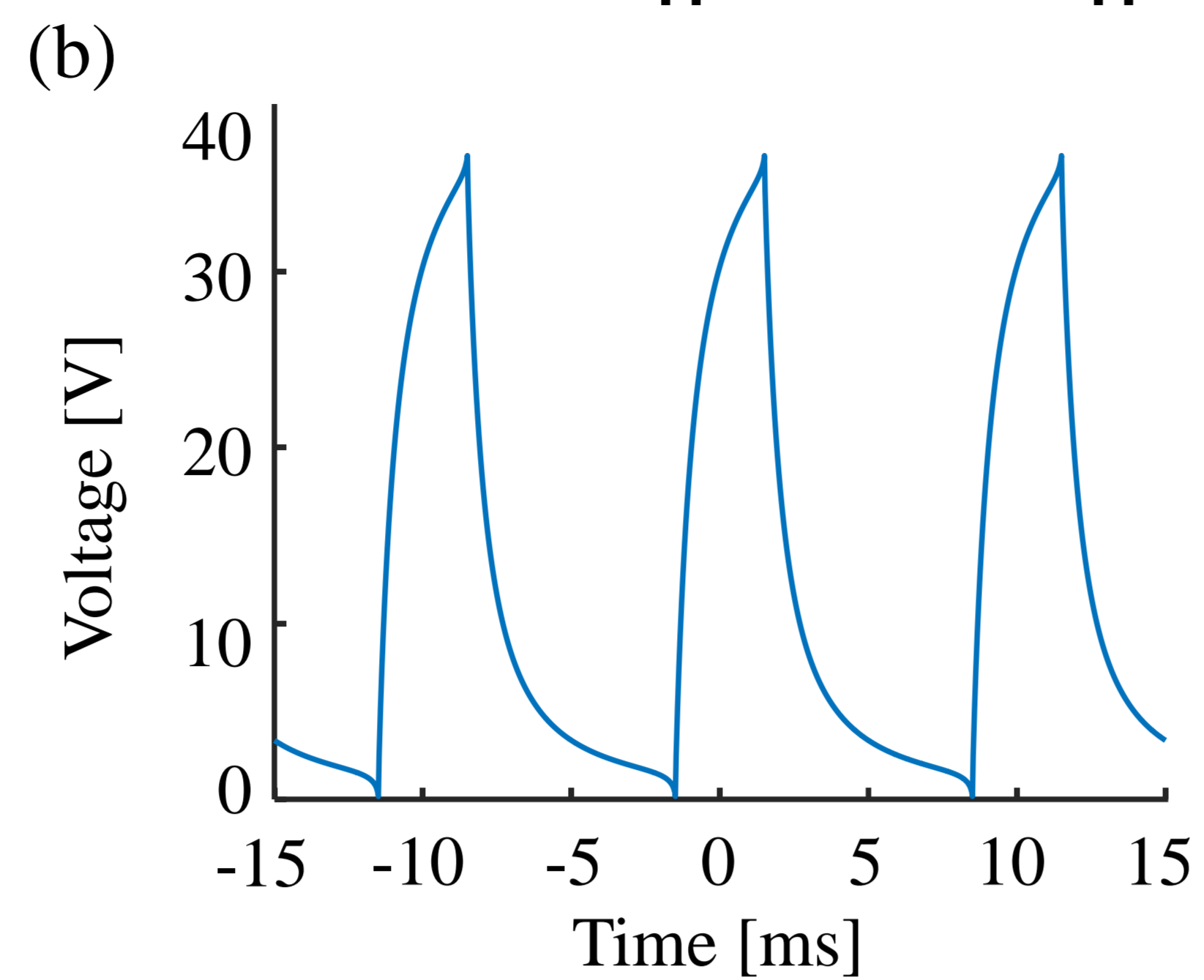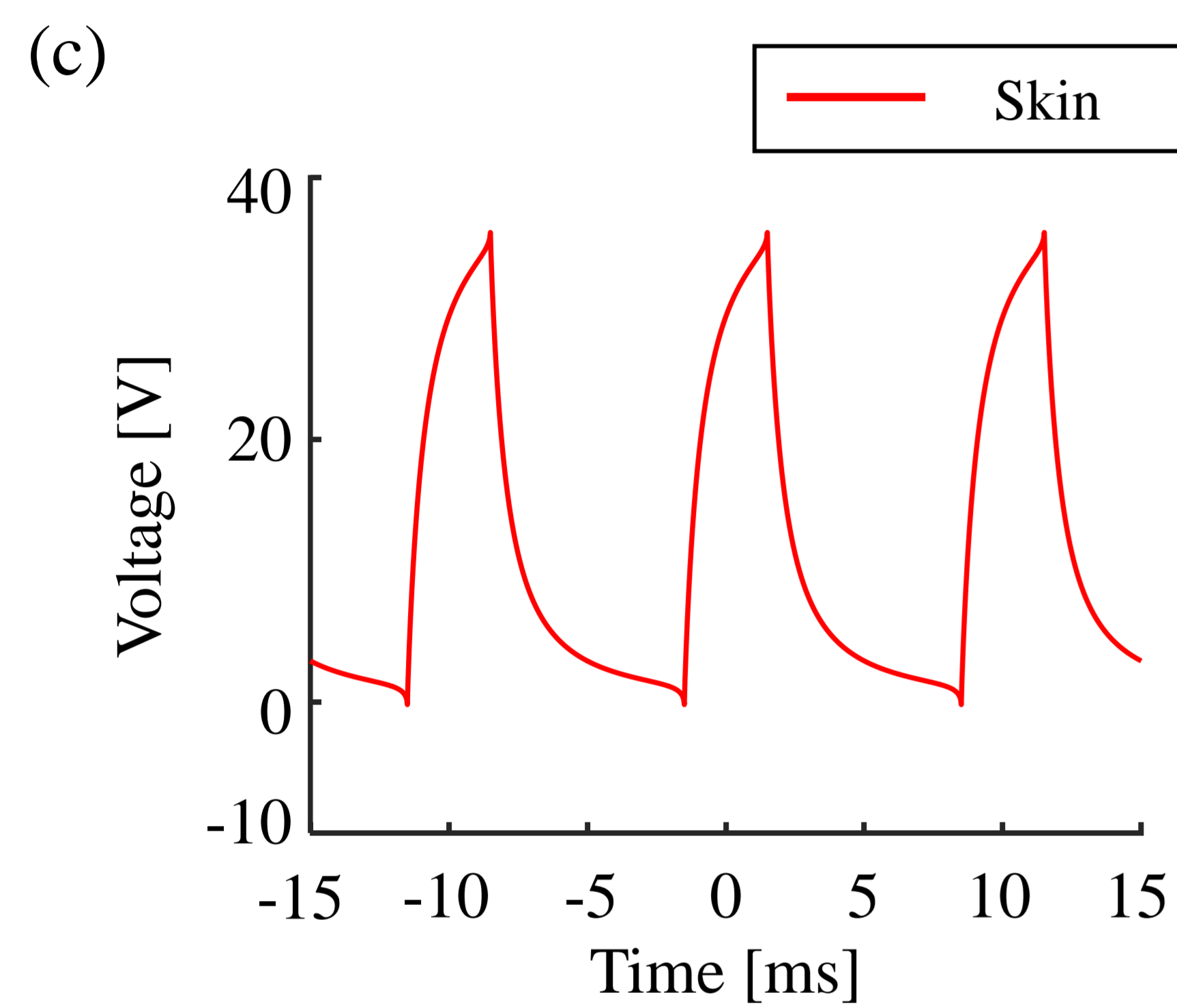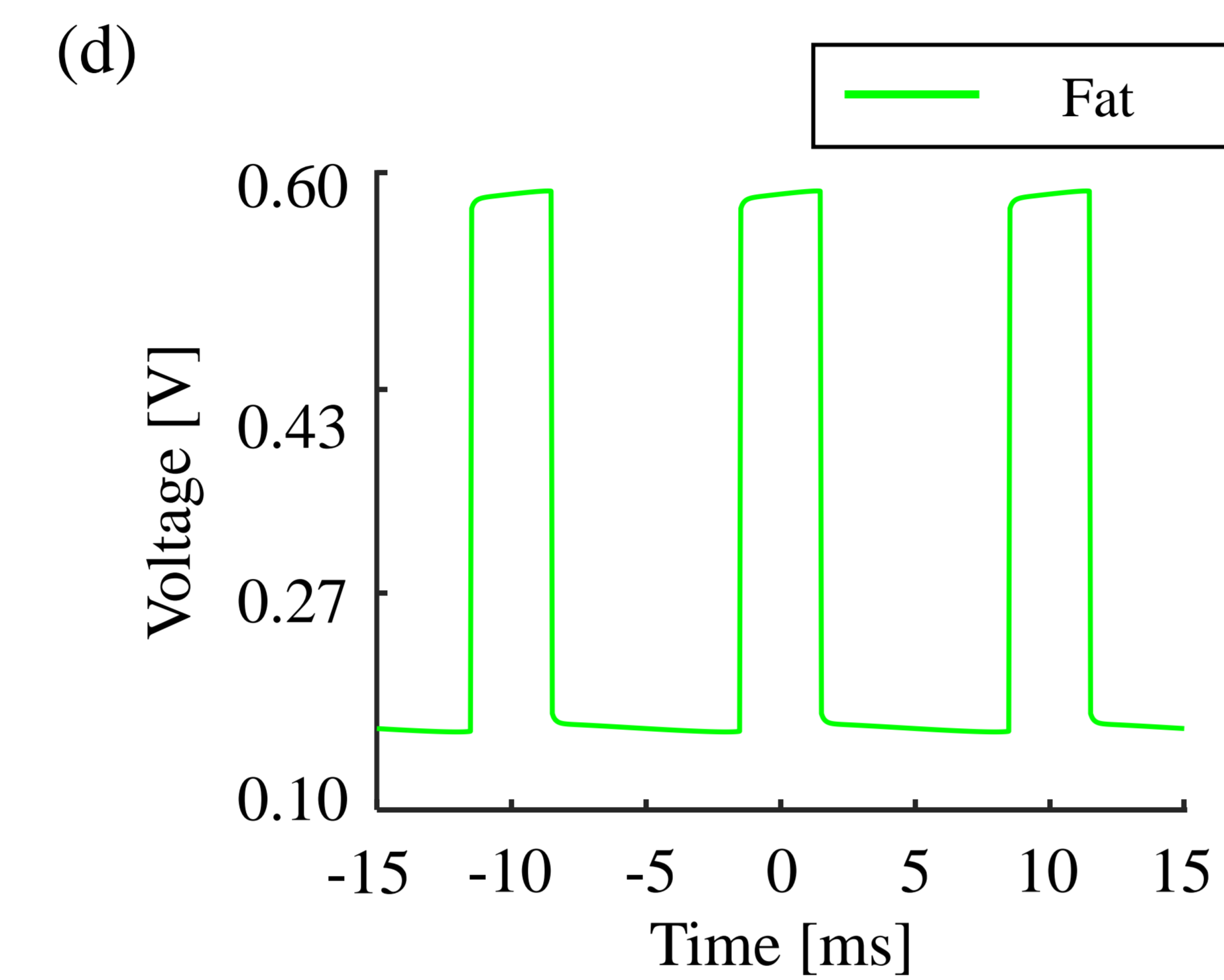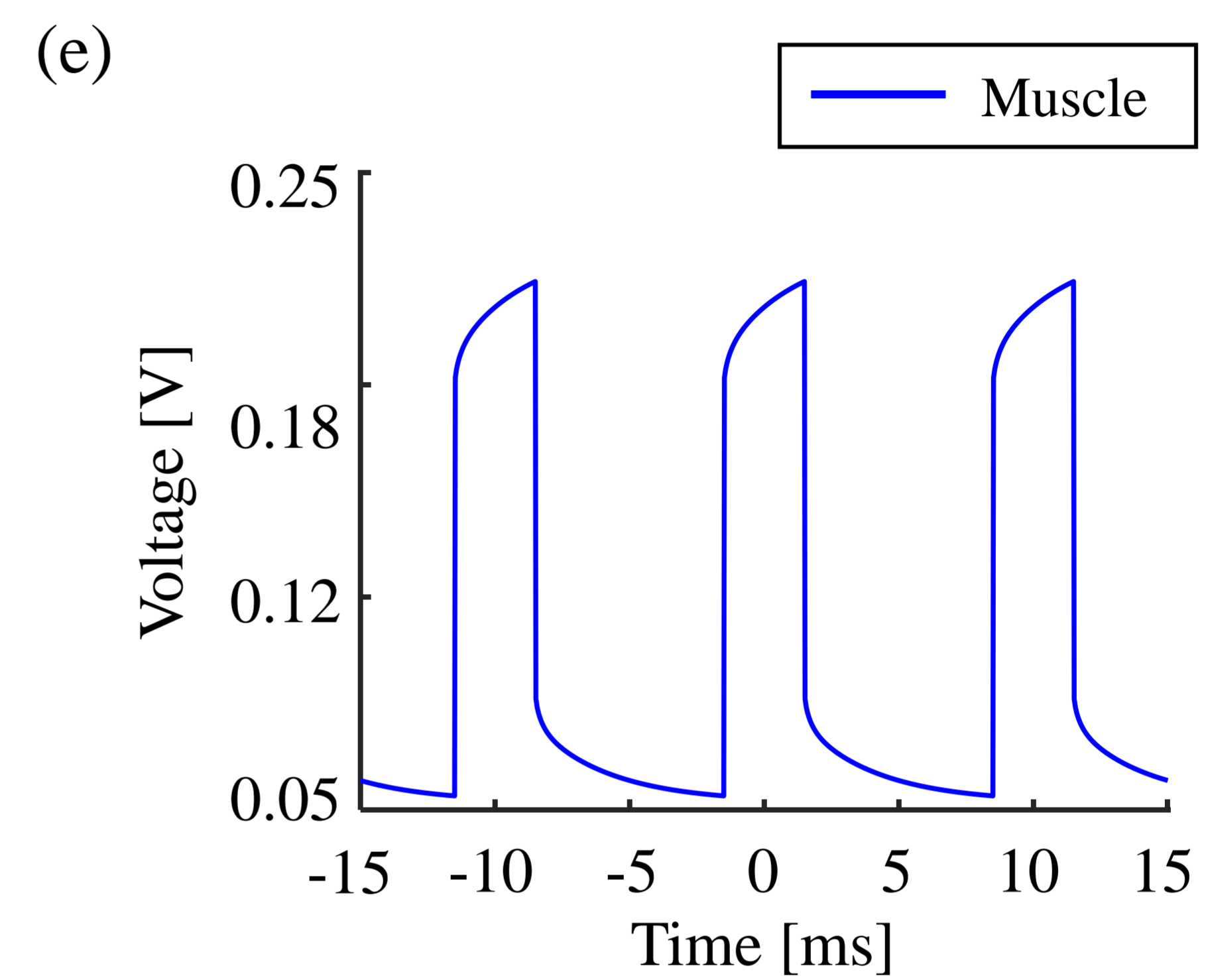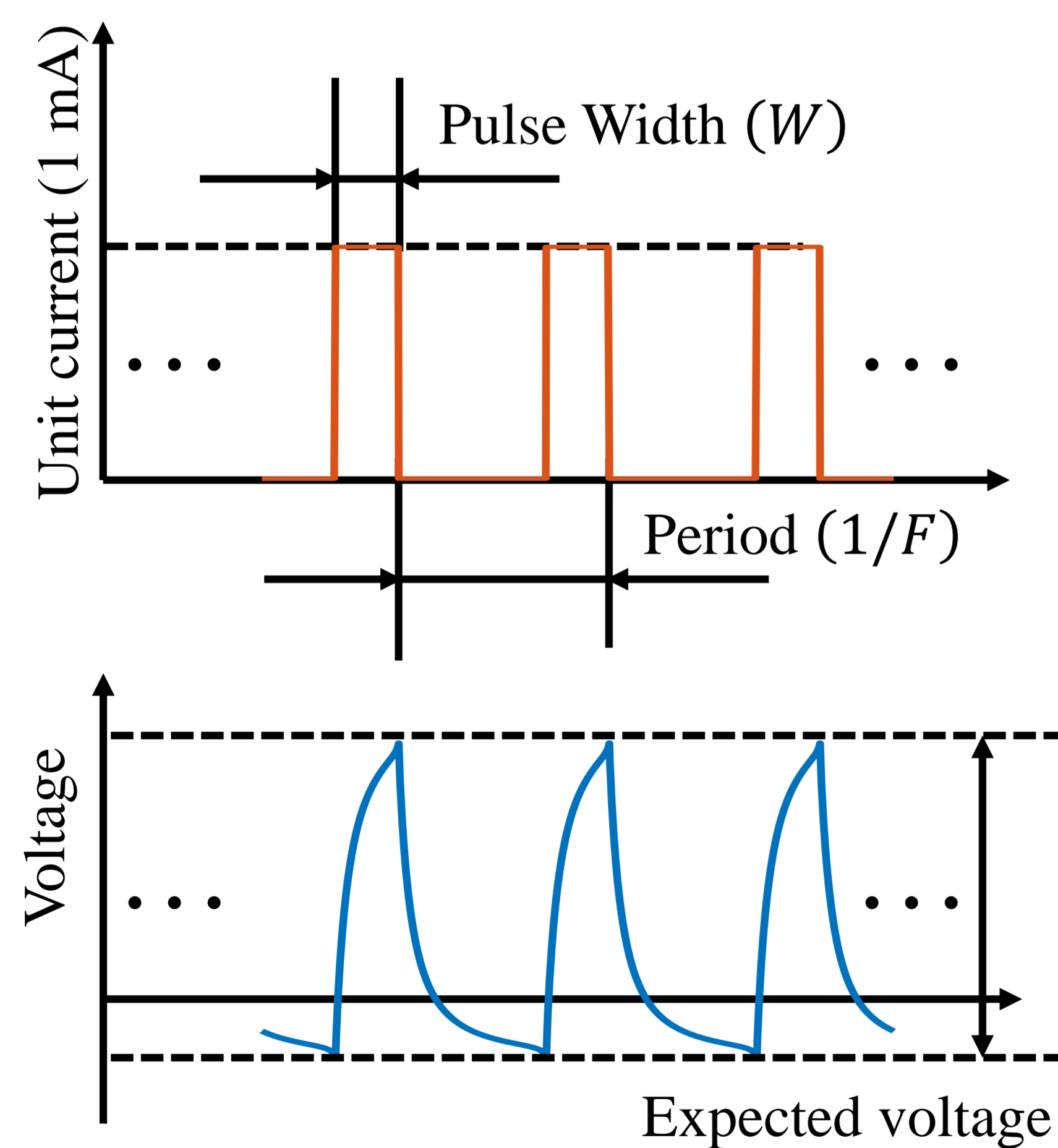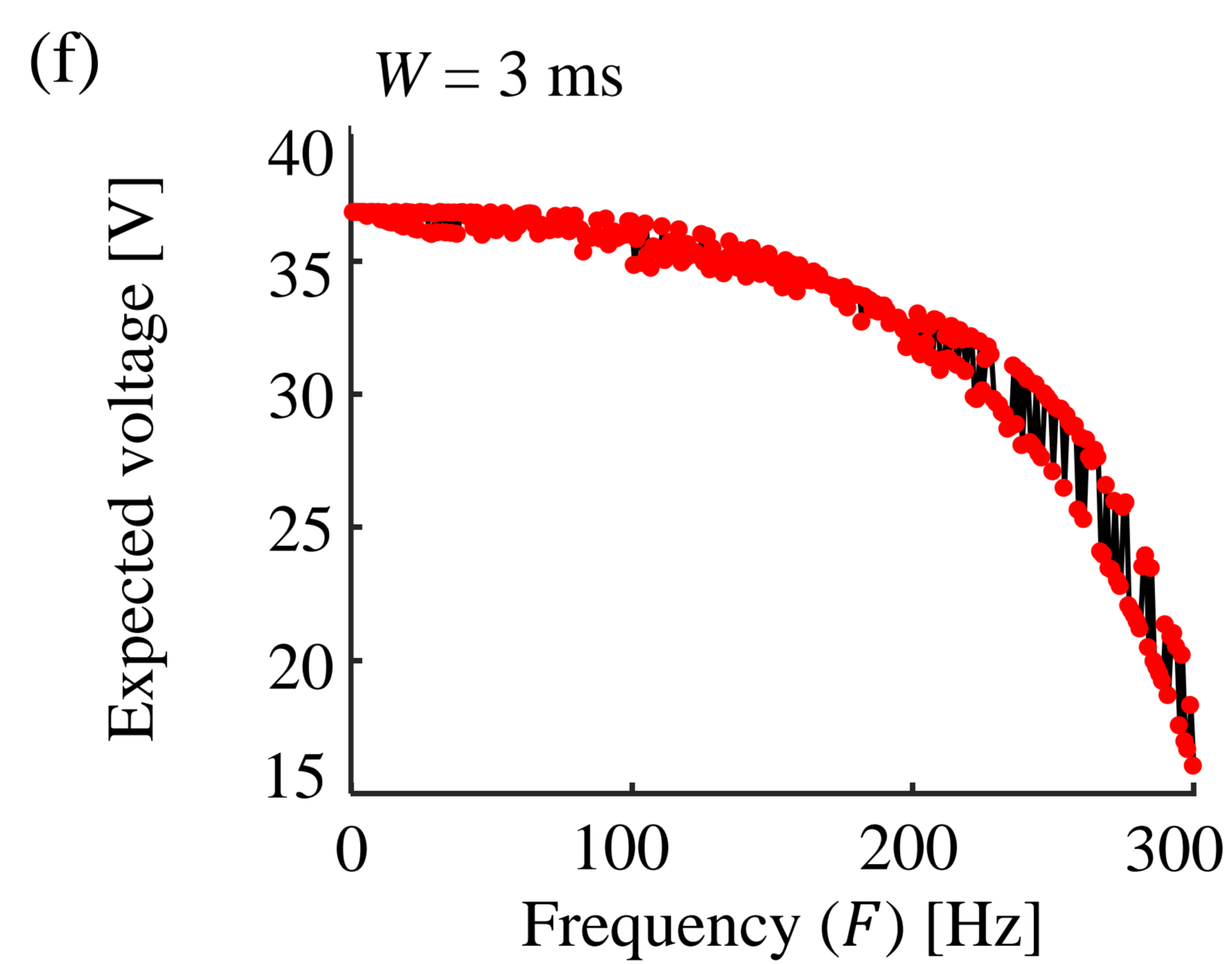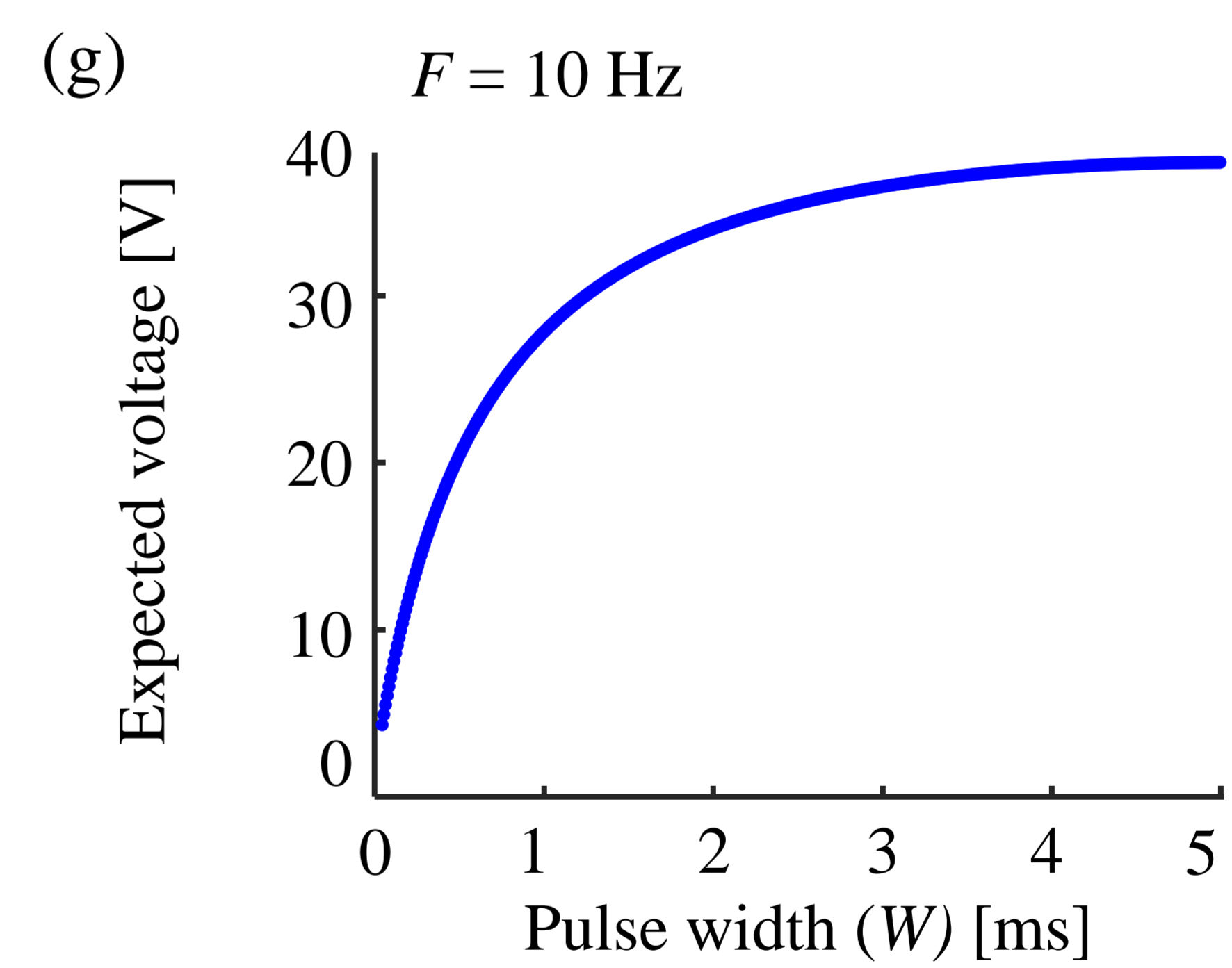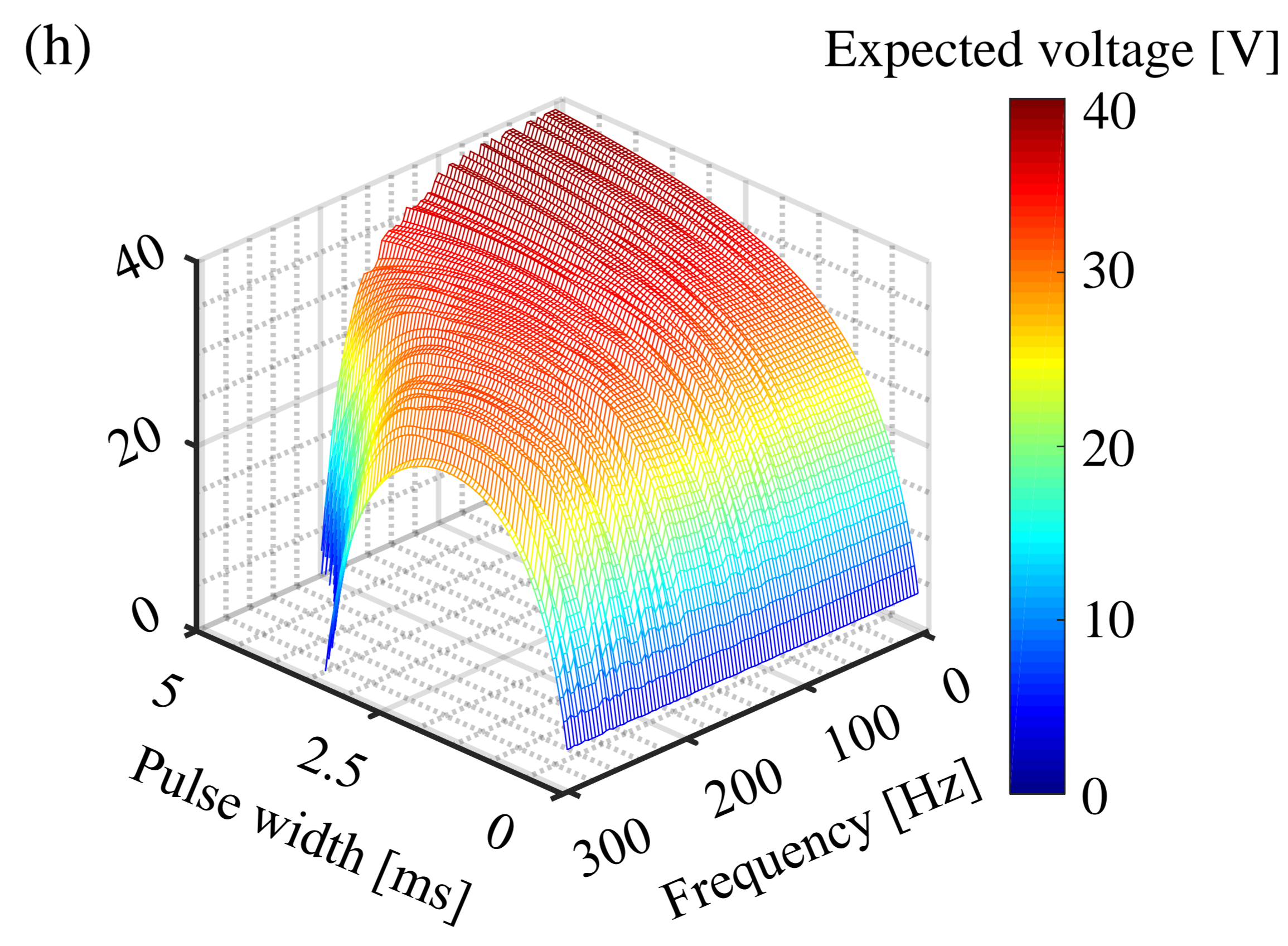

Supplement: Supplementary file 2 — Additional file 2 Figure S1. Electrical responses of the forearm impedance network to current-driven pulse stimulation. Using the developed TPB-ECM, the forearm impedance network and electrical responses to current-driven TENS were analyzed. [file 42490_2021_54_MOESM2_ESM.pdf]

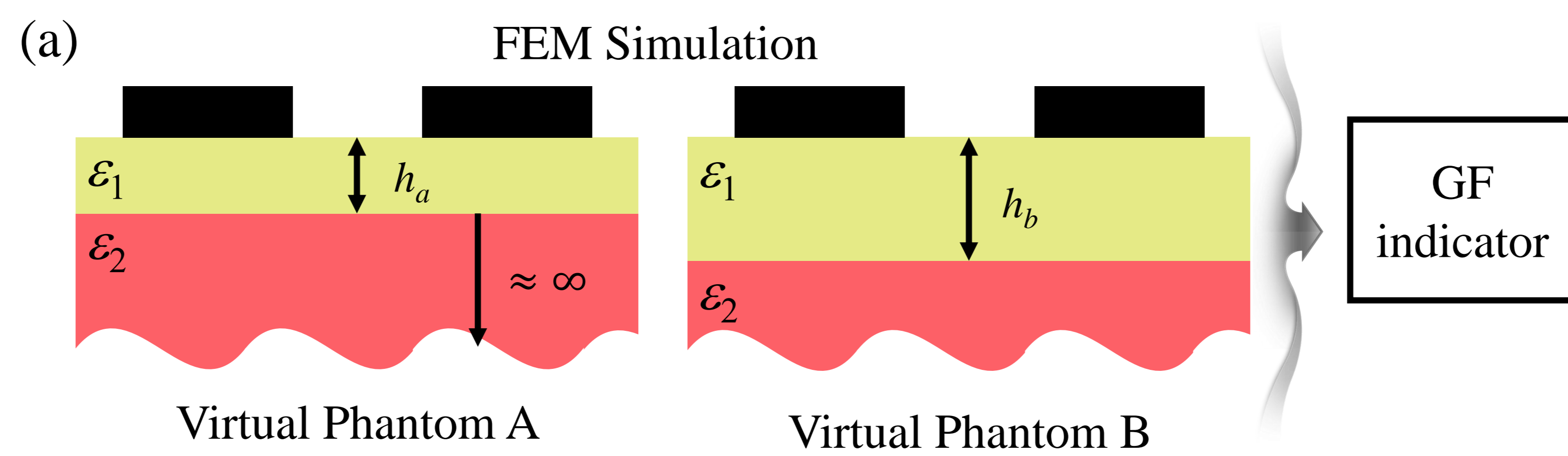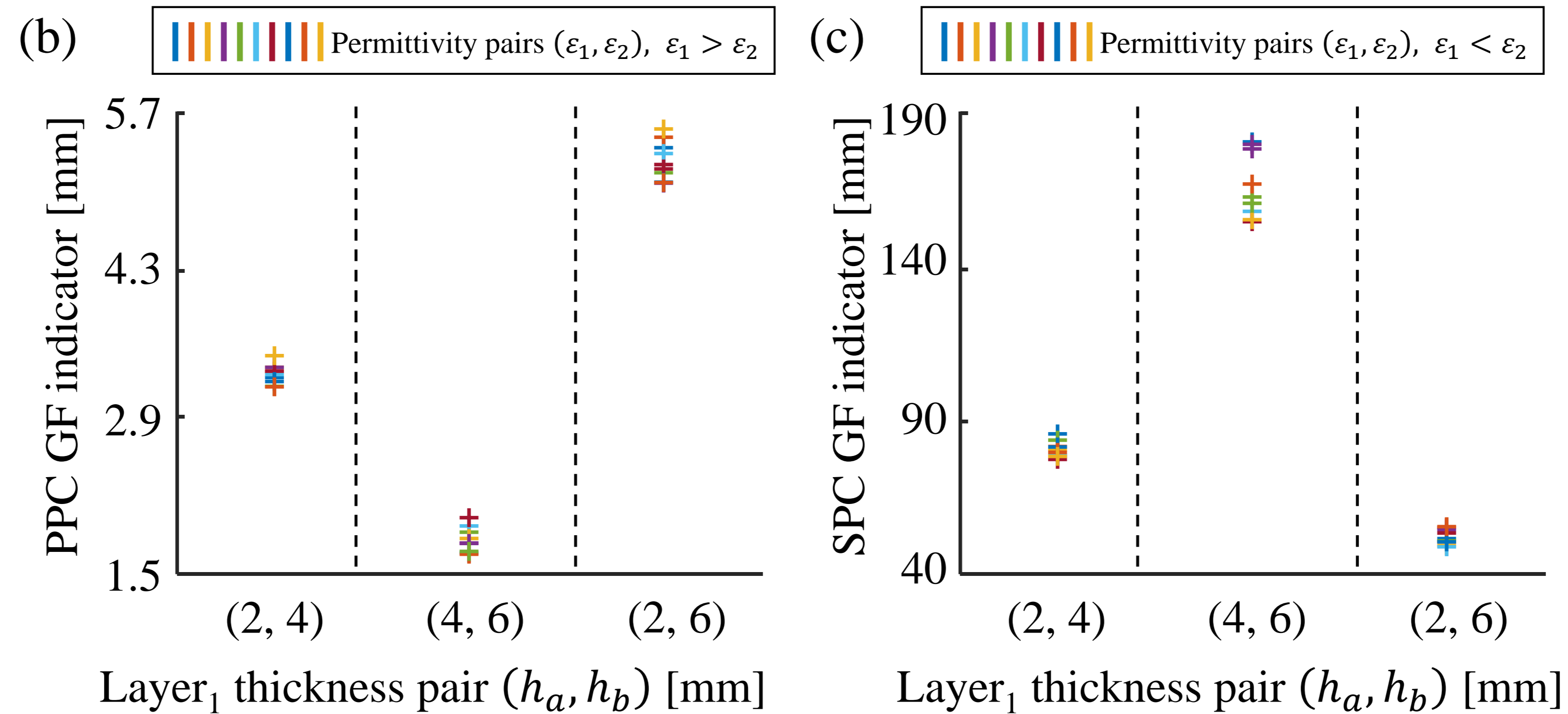

Supplement: Supplementary file 3 — Additional file 3 Figure S2. GF indicator calculated from in silico experiments using FEM simulator. a) PPC GF indicator, b) SPC GF indicator. [file 42490_2021_54_MOESM3_ESM.pdf]

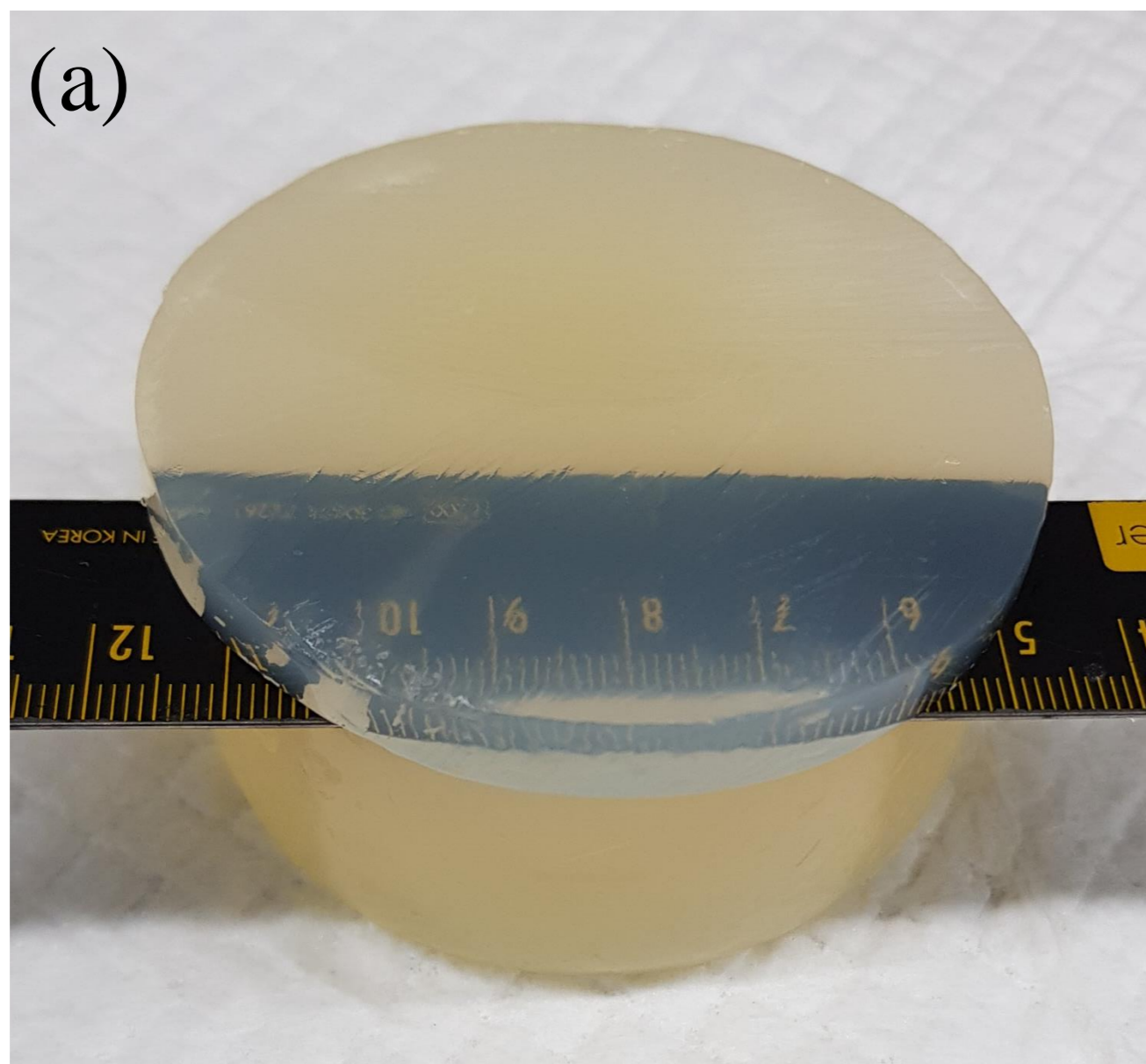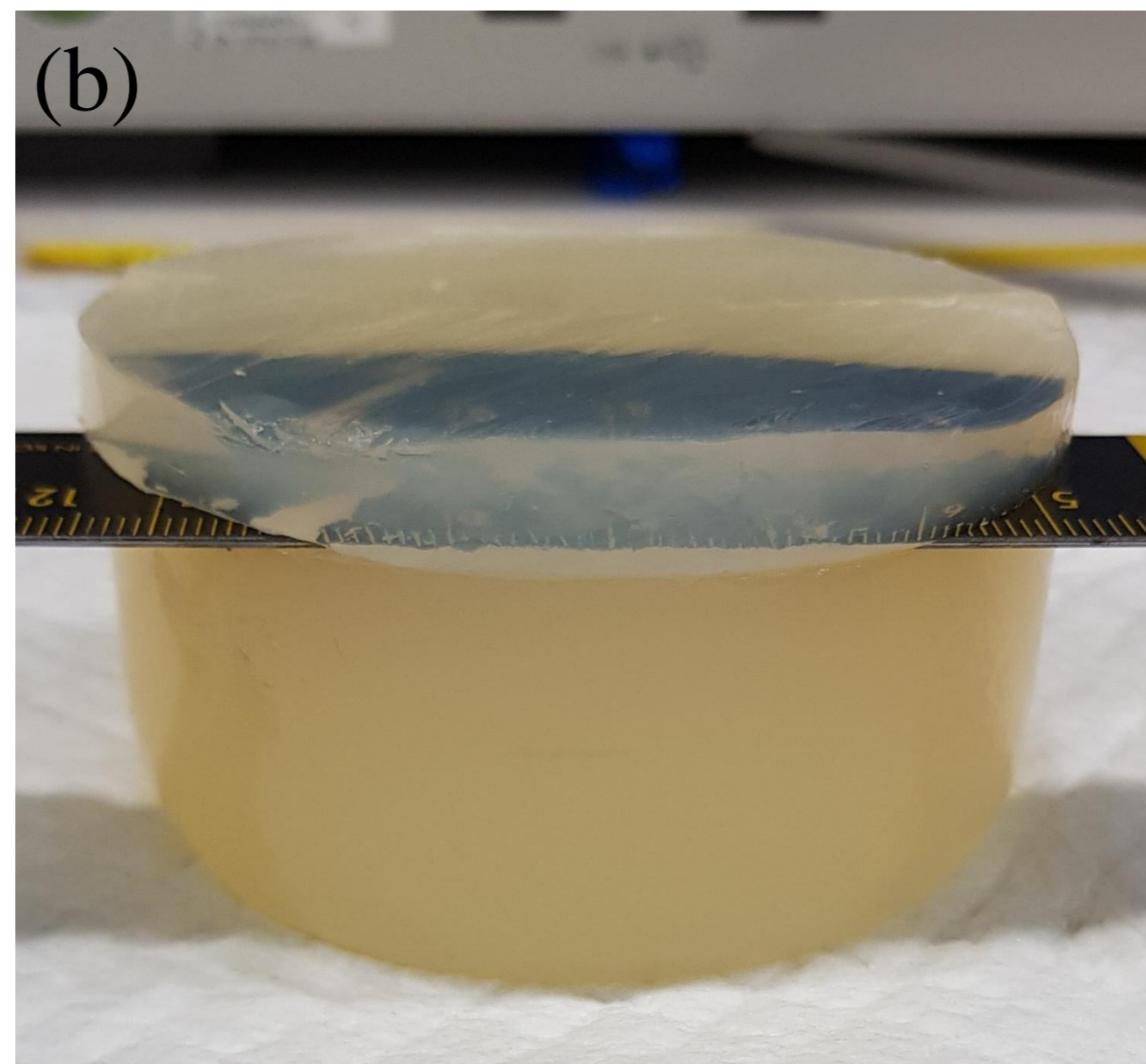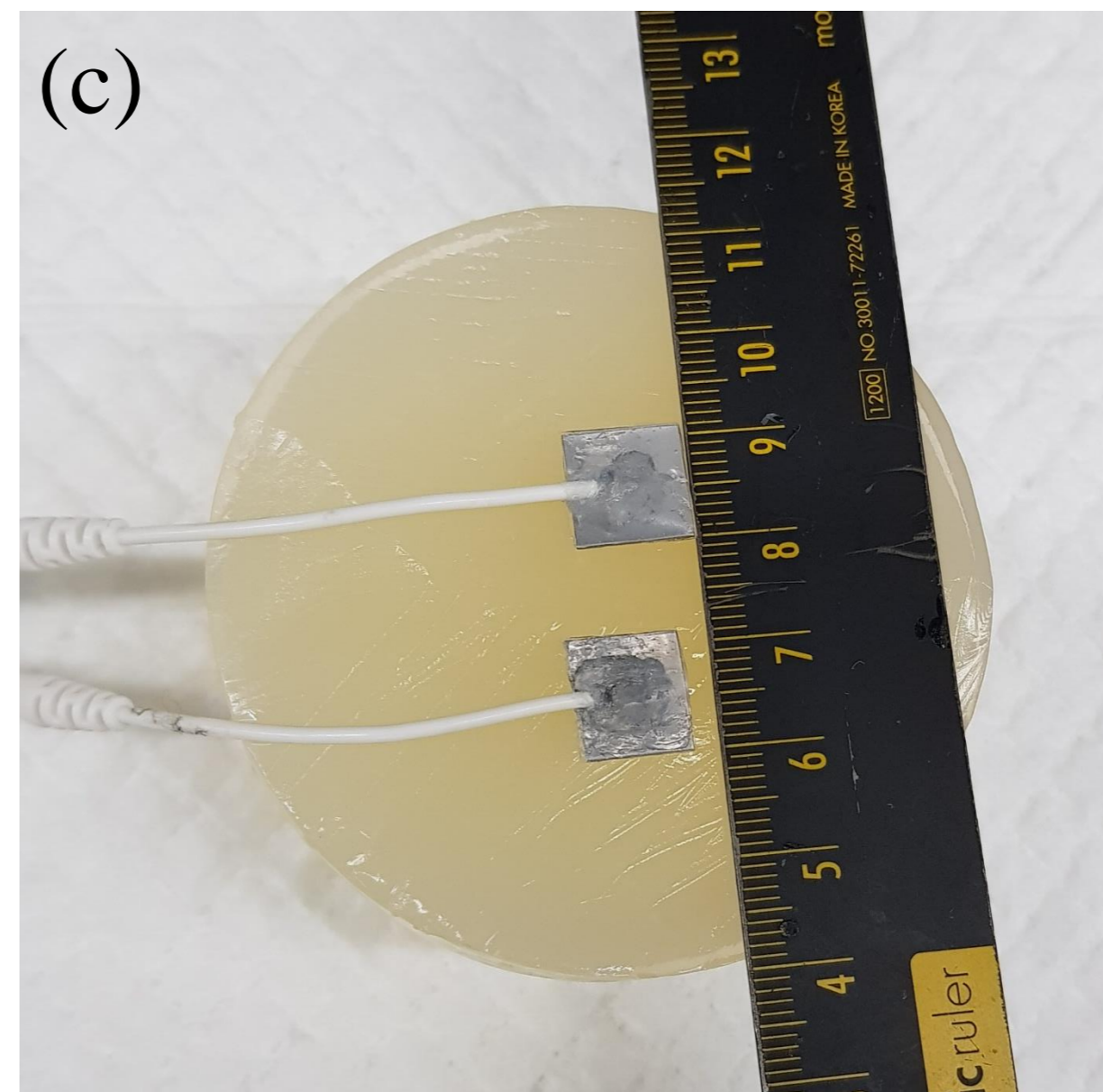

Supplement: Supplementary file 4 — Additional file 4 Figure S3. Pictures of in vitro agar experiments for TPB-ECM validation. a) &b) The double-layered agar phantom, c) representative view of the placement of measurement electrodes on the double-layered agar phantom. [file 42490_2021_54_MOESM4_ESM.pdf]
